# Population-Based Geospatial and Molecular Epidemiologic Study of Tuberculosis Transmission Dynamics, Botswana, 2012–2016

**DOI:** 10.3201/eid2703.203840

**Published:** 2021-03

**Authors:** Nicola M. Zetola, Patrick K. Moonan, Eleanor Click, John E. Oeltmann, Joyce Basotli, Xiao-Jun Wen, Rosanna Boyd, James L. Tobias, Alyssa Finlay, Chawangwa Modongo

**Affiliations:** University of Pennsylvania Partnership, Philadelphia, Pennsylvania, USA (N.M. Zetola, C. Modongo);; Centers for Disease Control and Prevention, Atlanta, Georgia, USA (P.K. Moonan, E. Click, J.E. Oeltmann, J. Basotli, X.-J. Wen, R. Boyd, J.L. Tobias, A. Finlay)

**Keywords:** tuberculosis, TB, tuberculosis and other mycobacteria, Mycobacterium tuberculosis, bacteria, respiratory infections, population-based analysis, geospatial analysis, molecular epidemiology, transmission dynamics, Kopanyo study, Botswana

## Abstract

Tuberculosis (TB) elimination requires interrupting transmission of *Mycobacterium tuberculosis*. We used a multidisciplinary approach to describe TB transmission in 2 sociodemographically distinct districts in Botswana (Kopanyo Study). During August 2012–March 2016, all patients who had TB were enrolled, their sputum samples were cultured, and *M. tuberculosis* isolates were genotyped by using 24-locus mycobacterial interspersed repetitive units–variable number of tandem repeats. Of 5,515 TB patients, 4,331 (79%) were enrolled. Annualized TB incidence varied by geography (range 66–1,140 TB patients/100,000 persons). A total of 1,796 patient isolates had valid genotyping results and residential geocoordinates; 780 (41%) patients were involved in a localized TB transmission event. Residence in areas with a high burden of TB, age <24 years, being a current smoker, and unemployment were factors associated with localized transmission events. Patients with known HIV-positive status had lower odds of being involved in localized transmission.

Tuberculosis (TB) was declared a public health emergency by the World Health Assembly in 2014, and the development of an ambitious global strategy to eliminate TB by 2035 soon followed (*1*,*2*). Five years later, progress toward elimination remains slow (*3*), in part because of the lack of effective interventions to interrupt the cycle of TB transmission (*4*). Ongoing transmission is the main driver of TB prevalence in high-burden communities (*5*,*6*). Historically, TB was believed to be the result of prolonged exposure to infectious TB patients, such as household contacts (*7*). More recently, molecular epidemiologic studies highlighted the possible role of casual exposures in the community (*8*,*9*). TB incidence and rates of TB transmission vary considerably across communities and might be dependent on high-risk behaviors, social determinates of disease (e.g., malnutrition, overcrowding, poverty), population dynamics, and transmission venues (*10*–*12*). Accordingly, interest has been renewed in increasing yield and cost-effectiveness of geographically targeted interventions.

Incremental progress toward elimination is possible with careful evidence-guided policy development, planning, and implementation. The design of effective, targeted TB interventions should be tailored to local epidemiology and program performance. In this population-based study, named the Kopanyo Study, we used a multidisciplinary approach combining classic epidemiologic approaches (i.e., relying on the behavioral, clinical, demographical, geospatial, social, and temporal characteristics of cases) with mycobacterial genetics to describe TB transmission in 2 large districts in Botswana.

## Methods

### Study Objective and Design

Kopanyo means “people gathering together” in the local Tswana language in Botswana. Consistent with this name, the overarching goal of the Kopanyo Study was to use geospatial analysis and patient interviews to define TB transmission networks and locations of TB transmission in 2 districts in Botswana, a country characterized by high rates of TB and HIV. More precisely, we aimed to describe and compare the clinical and microbiological characteristics of TB patients given a diagnosis in Gaborone and Ghanzi districts; describe and compare the spatial clustering and genotype clustering of patients and strains across and within districts; and determine the factors associated with genotype clustering, spatial clustering, as well as combined genotype and spatial clustering across and within districts. The design and procedures of this population-based, prospective study have been described in detail elsewhere (*8*,*13*).

### Setting

**Figure 1:**
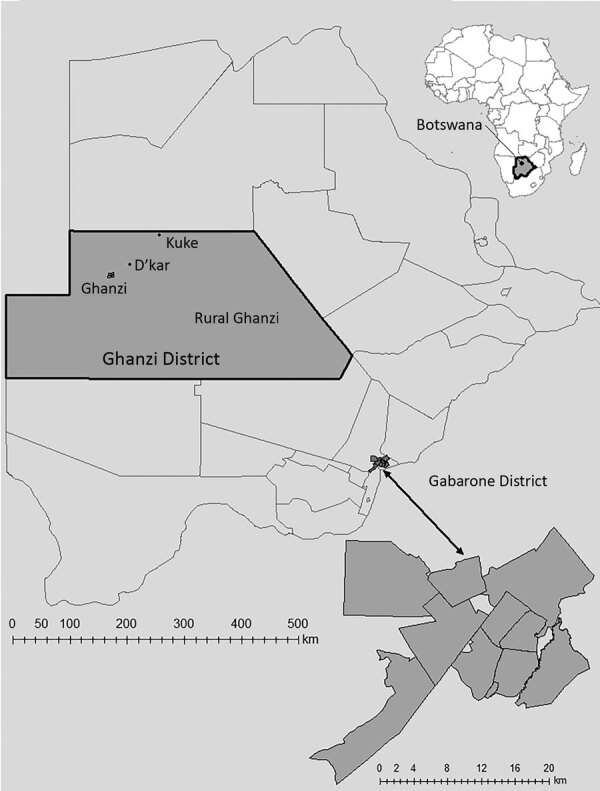
Catchment areas for population-based geospatial and molecular epidemiologic study of tuberculosis transmission dynamics, Botswana, 2012–2016 (Kopanyo Study). The 2 catchment areas are outlined in black. The neighborhoods within the Gaborone district (A–K, enlarged at bottom right) and Ghanzi district (W, DK, KU, and Y) are shown in gray. Inset map shows location of Botswana in Africa.

Botswana is an economically and politically stable sub-Saharan country that has a universal healthcare system for its citizens. We recruited participants from 2 distinct geographic areas: the capital city and surrounding suburbs, Gaborone district; and the rural district of Ghanzi (Figure 1). The study sites were purposefully selected because they were believed to represent disparate populations in Botswana in terms of demographic, environmental, epidemiologic (i.e., HIV prevalence, TB prevalence) and socioeconomic characteristics. With a population of 354,380 persons, Gaborone is the largest and most crowded urban area in the country. In 2013, at the start of this study, 17% of the general population in Gaborone was estimated to be living with HIV (*14*). Annual TB rates in Gaborone ranged from 440 to 470 cases/100,000 population during the 5 years before study implementation; ≈70% of TB patients were co-infected with HIV (*15*).

In contrast, Ghanzi is a rural district in northwestern Botswana, and most of the 44,100 persons in this district live in congregate housing; the town of Ghanzi has a population of 12,179 persons. Most of the population in the district is of San ethnicity. The San kept a traditionally hunter-gatherer lifestyle until early 1990s, when they were forced to transition to farming as a result of government-mandated modernization programs. Since then, most San live in large and crowded private freehold farms most of the year. For short periods during the year (ranging from days to several weeks), they transition through mid-size villages and the town of Ghanzi. Migration between farms and villages in the district is the norm and it is seasonal. However, because of cultural and geographic reasons there is little migration outside the district. Thus, despite major rotational migration between villages and farms, the community remains highly insular. Altogether, these unique cultural and social conditions contribute to the higher rates of TB transmission in Ghanzi. Over the past 2 decades, the TB notification rate in Ghanzi district has consistently been the highest in the country (722 cases/100,000 persons) (*14*,*15*). In 2013, the proportion of the general population in Ghanzi estimated to be living with HIV (17%) was similar to that for Gaborone; however, only 36% of TB patients are co-infected with HIV (*14*).

### Recruitment

Participants were enrolled during August 2012–March 2016. All patients given a diagnosis of TB were eligible for enrollment. Participants were recruited from TB clinics and directly observed treatment centers in greater Gaborone (n = 24) and Ghanzi District (n = 6). Patients receiving TB treatment for >14 days before study screening, incarcerated persons, or those who did not consent were excluded from the study.

### Data Sources, Measurements, and Variables

Behavioral, clinical, and demographic information were obtained by medical record abstraction and standardized interview at enrollment. Primary residential address, work place address at diagnosis, and address of social gathering venues of patients were obtained through patient interview. All addresses were verified by site visit geotagging, or through a reference layer created by manually relocating addresses in satellite imagery by using OpenStreetMap (http://www.openstreetmap.org) (*16*), Google Maps, and ArcGIS (Environmental System Research Institute, https://www.esri.com) online geocoding services. WGS 84 projection system latitude and longitude coordinates (with 1.1-m precision) were exported for each address. Botswana population and housing data was used to define geographic boundaries and enumerate localized populations necessary for TB incidence rates (*17*). We defined high-burden geographic areas if the estimated annualized TB incidence was >305 TB patients/100,000 persons, which is the estimated national TB incidence rate at the start of the study period.

HIV status was determined for all enrolled participants. Following national guidelines, we offered HIV testing to all participants who did not have documented HIV test results or had negative test results from >12 months before enrollment. Patients were asked to report the average number of alcoholic beverages consumed on the same occasion in the previous 30 days, the number of days consuming alcohol in the previous 30 days, and if they currently smoke tobacco. We defined excessive alcohol consumption as a self-report of >5 drinks on the same occasion or drinking on >5 days within the previous 30 days (*18*). Venues for social gathering were classified as alcohol-related (e.g., bars, liquor stores, pubs, shebeens), places of work, places of worship (e.g., churches, mosques, temples), and healthcare facilities.

### Sputa Collection and Laboratory Methods

At least 1 expectorated sputum sample was obtained from each enrolled patient. Patients unable to produce enough sputum or high-quality sputum underwent inhaled nebulized hypertonic saline solution induction. Sputa were decontaminated by using the N-acetyl-L-cysteine and NaOH method with a final concentration of 1% NaOH, and then inoculated into 1 Mycobacterial Growth Indicator Tube (MGIT; Becton Dickinson, https://www.bd.com). MGIT cultures were incubated at 35°C–37°C in the MGIT960 instrument (Becton Dickinson) for <6 weeks. MGIT cultures scored as positive were examined by microscopy and Ziehl-Neelsen staining to identify acid-fast bacilli. SD. The Bioline TB Ag MPT64 Rapid Test (Abbott, https://www.globalpointofcare.abbott/en/product-details/sd-bioline-tb-ag-mpt64-rapid.html) was used to identify the *M*. *tuberculosis* complex. Cultures positive for acid-fast bacilli but with negative TB Ag MPT64 results were classified as nontuberculous mycobacteria. Cultures with evidence of both *Mycobacterium* species and other potential contaminating species were redecontaminated by using the standard method described above. Drug susceptibility testing (DST) for first-line anti-TB drugs was performed by using MGIT DST. Susceptibility was set at 0.1 μg/mL for isoniazid and 1.0 μg/mL for rifampin. We used DST with Lowenstein-Jensen medium in instances for which MGIT DST results were not available.

### *M. tuberculosis* Genotyping

The first culture isolate per patient was genotyped (Genoscreen, https://www.genoscreen.fr) by using 24-locus mycobacterial interspersed repetitive units–variable number of tandem repeats (MIRU-VNTR) and standardized methods (*19*). MIRU-VNTR results with >1 copy number at >1 loci (i.e., double alleles) as seen with mixtures of different clonal subpopulations, or with missing or indeterminate copy number at any locus, were considered noninterpretable for cluster assignment and were excluded from analysis (*20*). Two or more patient isolates that had valid, complete, and matching MIRU results were classified as a genotype cluster.

### Localized Transmission Events

We used SaTScan (https://www.satscan.org) to identify geographic areas with a larger-than-expected rate of unique genotype clusters. We also used data for all other culture-positive TB patients reported during the study as the background rate (*20*–*22*). In brief, all individual MIRU-VNTR results were assigned to the corresponding geocoordinates of the patient’s residence. Each unique MIRU-VNTR result was then scanned separately, applying a purely spatial analysis, in which the number of events in an area was assumed to be Poisson distributed to generate circular zones of various sizes up to a maximum radius of 50 km. A log-likelihood ratio was calculated for each zone in comparison with all possible zones, with the maximum likelihood ratio representing the zone most likely to identify statistically significant spatial concentrations for each MIRU-VNTR result. Thus, by definition, localized transmission was characterized by genotypic and spatial clustering. A Monte Carlo simulation with 9,999 repetitions was used to determine the distribution of the scan statistic under the null hypothesis of spatial randomness; significant spatial clusters were chosen by using an α of p<0.05. No duplicative case counting occurred. The purpose of the spatial scan was to characterize each patient (based on residence) for a dichotomous outcome: member of a localized transmission event or not.

### Statistical Methods

Annualized TB incidence per 100,000 persons and 95% CIs, assuming a Poisson distribution, were calculated for local geographies on the basis of the number of cases enrolled from each geography divided by the population estimates for each geography annualized to the duration of the study period. Estimates were assigned to the geographic centroid in ArcGIS. Isopleth cartographic images were produced by using a raster layer interpolated with inverse distance weighting (*21*). Differences in proportions between behavioral, clinical, and demographic variables by geographic location were assessed by using the χ^2^ test. Multivariable logistic regression analysis was conducted to assess the association of involvement in a localized transmission event (coded as a binary yes/no variable) and select variables by using adjusted odds ratios (aORs) that were significant at the 95% CIs. All variables statistically associated with the main outcome in bivariable analyses (p<0.1) were included in the multivariate model.

### Ethics

This study was approved by the Centers for Disease Control and Prevention Institutional Review Board; the Health Research and Development Committee, Ministry of Health and Wellness, Botswana; and the University of Pennsylvania Institutional Review Boards. Participants provided written informed consent.

## Results

### Patient Characteristics

**Figure 2:**
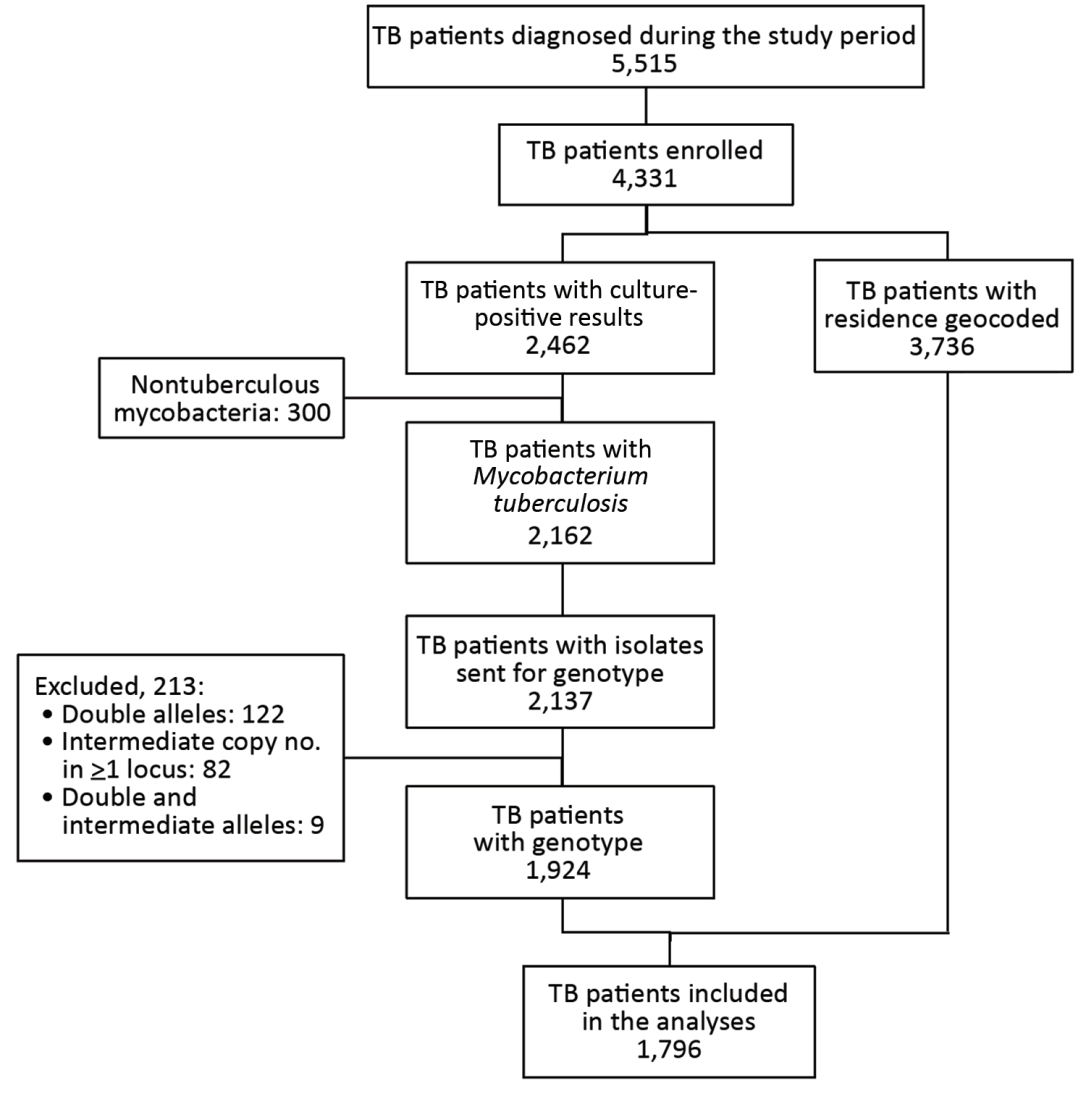
Flowchart of study enrollment for population-based geospatial and molecular epidemiologic study of TB transmission dynamics, Botswana, 2012–2016. TB, tuberculosis.

A total of 5,515 patients were given a diagnosis of TB during the study period; 4,331 (79%) were enrolled (Figure 2). Primary residence was geocoded and validated for 3,736 (86%) participants. Of these participants, 2,659 (71%) resided in Gaborone District, 723 (19%) resided in Ghanzi District, and 354 (10%) resided in other locations outside Gaborone or Ghanzi Districts. There were no significant differences in proportions with regards to sex (p = 0.116) and having valid MIRU results (p = 0.555) between the 3 locations (Table 1). However, there were differences in proportions for Gaborone, Ghanzi District, and other locations, respectively, regarding age (p<0.001), residence in a high-burden neighborhood (14% vs. 73% vs. 0%; p<0.001), excessive alcohol use (25% vs. 32% vs. 14%; p<0.001), current smoking (20% vs. 43% vs. 13%; p<0.001), unemployment (30% vs. 67% vs. 58%; p<0.001) history of incarceration (5% vs. 8% vs. 5%; p = 0.007), positive HIV status (66% vs. 37% vs. 64%; p<0.001), culture-positive TB (68% vs. 78% vs. 48%; p<0.001), and MDR TB at baseline (1% vs. 2 vs. 6%; p<0.001) (Table 1).

**Table 1:** Characteristics of enrolled patients by geography venues for population-based geospatial and molecular epidemiologic study of tuberculosis transmission dynamics, Botswana, 2012–2016*

Characteristic	No. (%) patients		
	Gaborone, n = 2,659	Ghanzi District, n = 723	Other, n = 354†
Sex			
M	1,501 (56.4)	403 (55.7)	181 (51.1)
F	1,158 (43.6)	320 (44.3)	173 (48.9)
Age, y			
<15	111 (4.2)	91 (12.6)	47 (13.7)
16–24	329 (12.4)	126 (17.5)	32 (9.0)
25–40	1,388 (52.)	269 (37.3)	134 (37.9)
41–64	760 (28.6)	185 (25.6)	113 (31.9)
≥65	71 (2.7)	51 (7.1)	28 (7.9)
Income, Pula			
None	855 (32.3)	536 (74.4)	223 (63.4)
<800	186 (7.0)	89 (12.4)	17 (4.8)
801–1,500	646 (24.4)	53 (7.4)	21 (6.0)
1,501–2,999	479 (18.1)	23 (3.2)	39 (11.1)
3,000–4,999	225 (8.5)	9 (1.3)	21 (6.0)
5,000–9,999	184 (7.0)	8 (1.1)	18(5.1)
>10,000	68 (2.6)	2 (0.3)	13 (3.7)
Resided in high-burden geographic area‡	370 (13.9)	530 (73.3)	NA
Excessive alcohol use§	661 (24.9)	230 (31.8)	51 (14.4)
Current smoker	517 (19.5)	307 (42.5)	45 (12.7)
Unemployed	760 (29.8)	421 (66.6)	178 (58.0)
History of incarceration	143 (5.4)	61 (8.4)	18 (5.1)
HIV status			
Positive	1,706 (66.2)	259 (37.4)	221 (63.5)
Negative	872 (30.7)	434 (60.0)	127 (35.9)
Unknown	81 (3.1)	30 (2.6)	6 (0.6)
CD4+ cells/mm^3^ at diagnosis, if HIV positive			
0–199	436 (44.7)	62 (30.8)	83 (54.2)
200–499	383 (39.2)	85 (42.3)	46 (30.1)
≥500	157 (16.1)	54 (26.9)	24 (15.7)
Previous TB episode	444 (16.7)	219 (30.3)	90 (25.4)
Culture positive	1,449 (68.1)	435 (78.0)	125 (47.9)
MDR TB at baseline	27 (1.1)	14 (2.1)	17 (5.8)

*Values are no. (%). MDR TB, multidrug-resistant tuberculosis; NA, not applicable; TB, tuberculosis. †Includes patients with primary residence outside Gaborone and Ghanzi. Missing residential address, n = 595. ‡Residing in a geographic area with an estimated annualized TB incidence >305 patients/100,000 persons. Excludes 407 patients with culture results but no geocoded residence. §Five or more drinks/session or drinking on >5 days/month.

### Geographic Distribution of TB

The estimated annualized TB incidence for the overall study population was 306 TB patients/100,000 persons (95% CI 228–339) (Table 2). The incidence rate varied considerably by geography, ranging from 66 (95% CI 44–99) TB patients/100,000 persons in the suburban areas of Gaborone to 1,140 (95% 836–1,556) TB patients/100,000 persons in remote, rural villages of the Ghanzi District. The degree of heterogeneity was more pronounced in Gaborone than in Ghanzi District. For example, a 7.3-fold difference in annualized incidence was found between the highest (location A) and lowest (location J) burden areas in Gaborone. This difference was 1.3 fold in Ghanzi District (highest location W and lowest location KU). In this context, we observed that certain neighborhoods contributed disproportionally to the district-level burden of TB. Some locations that had the lowest TB prevalence had the highest number and proportion of patients co-infected with HIV (Table 1).

**Table 2:** Demographic and clinical characteristics by geographic area venues for population-based geospatial and molecular epidemiologic study of tuberculosis transmission dynamics, Botswana, 2012–2016*

Area	Populati­on†	Size, km^2^	Population density†	No. enrolled TB patients	No. who had TB (95% CI)‡	No. (%) enrolled HIV-positive patients	No. *Mycobacterium tuberculosis* genotypes§
Gaborone							
A	19,143	1.4	13,973	370	483 (438–536)	245 (66.2)	107
B	32,805	21.8	1,505	183	139 (121–161)	109 (59.6)	71
C	59,100	16.0	3,694	591	250 (231–271)	379 (64.1)	168
D	51,190	53.9	950	574	280 (259–305)	364 (63.4)	170
E	34,262	39.5	867	249	182 (161–206)	165 (66.3)	97
F	71,957	27.5	2,617	388	135 (122–149)	243 (62.6)	148
G	28,639	17.9	1,600	139	121 (104–144)	79 (56.8)	64
H	12,094	24.6	492	71	147 (117–185)	54 (76.1)	26
I	7,677	77.7	99	28	91 (63–132)	17 (60.7)	14
J	8,729	88.2	99	23	66 (44–99)	17 (73.9)	7
K	14,104	72.4	195	43	76 (58–105)	34 (79.1)	16
Ghanzi District							
W	12,179	45.8	266	419	860 (783–945)	158 (37.7)	80
DK	1,668	9.7	172	73	1,094 (874–1,369)	17 (23.3)	16
KU	833	7.5	111	38	1,140 (836–1,556)	16 (42.1)	9
Y	2,203	1,607	1.3	193	NA	68 (35.2)	45
Other location	354	NA	NA	354	NA	221 (62.4)	89
Not geocoded	NA	NA	NA	595	NA	366 (61.5)	100
Total	354,380	NA	NA	4,331	306 (228–339)	2,552 (58.9)	686

*NA, not applicable; TB, tuberculosis. †Persons residing/km^2^. Based 2011 Population and Housing Census. Statistics Botswana. ‡TB incidence/100,000 persons; 95% CIs assume a Poisson distribution);based on no. cases enrolled from each geography divided by the population estimates for each geography annualized to the duration of study period. §Rows are not mutually exclusive; strains might be found in multiple locations.

### Patient Isolate Characteristics

A total of 2,462 (56%) patients had >1 positive culture result; 2,162 (88%) were classified as *M. tuberculosis* complex, whereas 300 (12%) were classified as nontuberculous mycobacteria and were excluded. MIRU-VNTR results were available for 2,137 patient isolates. We excluded 213 (10%) patient isolates that had incomplete or noninterpretable genotyping results; this exclusion was described elsewhere (*23*,*24*). Thus, 1,924 patients were included in phylogenetic analysis. Among these patients, 128 had no residential geocoordinates, which resulted in 1,796 patients available for localized transmission analysis (Figure 2).

### Localized Transmission

**Figure 3:**
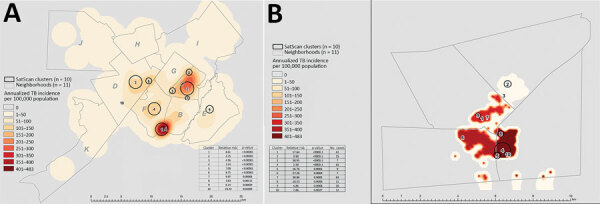
Annualized estimated TB incidence with the most probable localized transmission events superimposed, Botswana, 2012–2016. A) Gaborone; B) Ghanzi District. Shown are areas with most probable clusters identified by using spatial scan statistics (discrete Poisson) for 10 major clusters in Gaborone and Ghanzi (black circles). Data are superimposed on the inverse distance weighted map of annualized incidence of TB patients by neighborhood. TB, tuberculosis.

A total of 780 (43%) patients were members of localized transmission events. Among these patients, 537 (69%) resided in Gaborone, 241 (31%) resided in the Ghanzi District, and 2 (0.3%) resided elsewhere. Localized transmission was independently associated with younger age (<15 years of age, aOR 2.20, 95% CI 1.67–4.15; 16–24 years of age, aOR 1.41, 95% CI 1.05–1.89), residing in a high-burden neighborhood (aOR 2.75, 95 CI% 2.21–3.41), being a current smoker (aOR 1.71, 95% CI 1.38–2.11), and being unemployed (aOR 1.31, 95 CI% 1.08–1.59) (Table 3; Appendix Table). Patients who had a known HIV-positive status had lower odds of being a member of localized transmission (aOR 0.71, 95CI % 0.58–0.85). When we superimposed the SaTScan results over the interpolated with inverse distance-weighted TB incidence estimates, the spatial concentration for localized transmission coincided with higher TB incidence rates (Figure 3).

**Table 3:** Characteristics associated with localized tuberculosis transmission venues for population-based geospatial and molecular epidemiologic study of tuberculosis transmission dynamics, Botswana, 2012–2016*

Characteristic	Member of localized TB transmission, n = 780	Not a member of localized TB transmission, n = 1,016	Adjusted odds ratio (95% CI)
Sex			
M	436 (55.9)	555 (54.6)	1.05 (0.87–1.27)
F	334 (44.1)	461 (45.4)	
Age, y			
<15	26 (3.3)	18 (1.8)	**2.20 (1.67–4.15)**
16–24	157 (20.1)	170 (16.7)	**1.41 (1.05–1.89)**
25–40	418 (53.6)	558 (54.9)	1.14 (0.90–1.45)
41–64	158 (20.3)	241 (23.7)	Referent
>65	21 (2.7)	29 (2.9)	1.11 (0.61–3.00)
Resided in high-burden geographic area†	291 (37.1)	181 (17.8)	**2.75 (2.21–3.41)**
Excessive alcohol use‡	160 (20.6)	205 (20.2)	1.03 (0.82–1.30)
Current smoker	250 (32.1)	220 (21.7)	**1.71 (1.38–2.11)**
Unemployed	314 (41.6)	352 (35.3)	1.31 (1.08–1.59)
History of incarceration	49 (6.3)	56 (5.5)	1.15 (0.78–1.71)
HIV positive	366 (48.5)	570 (57.2)	**0.71 (0.58–0.85)**
Previous TB episode	156 (20.0)	178 (17.5)	1.17 (0.93–1.49)

*Values are no. (%). Bold indicates statistical significance at α = 0.05. Localized transmission defined by SaTScan (https://www.satscan.org)–identified geographic areas with a larger-than-expected rate of unique genotype clustering compared with all other culture-positive TB patients as the background rate; excludes 128 patients with valid genotype results and no residential address. TB, tuberculosis. †Residing in a geographic area with an estimated annualized TB incidence > 305 patients/100,000 persons. ‡Five or more drinks/session in the previous 30 d or drinking on >5 days in the previous 30 d.

### Self-Reported Social Gathering Venues

The proportion matched by TB genotype was significantly larger in Ghanzi District (80%) than in Gaborone (64%) and other locations (4%; p<0.001) (Table 4). A total of 494 (22%) patients resided at the same address as another patient; among these, 29% matched by TB genotype. The proportion matched by TB genotype was significantly larger in the Ghanzi District (50%) than in Gaborone (18%; p<0.001). A total of 605 (32%) patients reported the same place of worship as another patient; among these, 11% matched by TB genotype. The proportion matched by TB genotype was significantly larger in Ghanzi District (24%) than in Gaborone (9%; p = 0.002). A total of 585 (30%) reported the same alcohol-related venue as another patient; among these, 28% matched by TB genotype. The proportion matched by TB genotype was significantly larger in Ghanzi District (57%) than in Gaborone (16%; p<0.001).

**Table 4:** Self-reported social gathering venues for population-based geospatial and molecular epidemiologic study of tuberculosis transmission dynamics, Botswana, 2012–2016*

Venue	Total, n = 1,924			Gaborone, n = 1,524			Ghanzi District, n = 400	
	Naming same venue	Naming same venue and same genotype result†		Naming same venue	Naming same venue and same genotype result†		Naming same venue	Naming same venue and same genotype result†
Alcohol-related‡	585 (30.4)	164 (28.0)		409 (26.8)	65 (4.3)		171 (42.8)	98 (24.5)
Place of worship‡	605 (31.4)	64 (10.6)		540 (35.4)	49 (3.2)		62 (15.5)	15 (3.8)
Same residence§	494 (29.4)	123 (28.8)		337 (22.1)	60 (3.9)		126 (31.5)	63 (15.8)

*Values are no. (%). †Does not imply temporality. ‡Includes patients with residential location that is outside Gaborone or Ghanzi or that is unknown. §Missing residential address, n = 128.

## Discussion

Interrupting TB transmission is paramount for achieving TB elimination in high-burden settings. Accordingly, increasing interest exists on determining where, when, and among whom TB transmission occurs. Our study helps clarify factors fueling the TB epidemic in Botswana and highlights the necessity of understanding local epidemiology to design effective interventions aimed at interrupting TB transmission. We combined isolate genotype and spatial clustering as an indicator consistent with localized transmission. Although we acknowledge that some misclassification might occur with this approach, it enabled us to broadly consider geographic and individual characteristics that might be associated with localized transmission.

We found high incidence rates and substantial variation in TB incidence between neighborhoods and districts. Actual incidence rates are likely higher because we calculated estimates on the basis of numbers of enrolled patients, but not all persons with TB were enrolled in this study. Residing in a high-burden geographic area was associated with localized transmission, likely reflecting more cumulative exposures leading to more infections, reinfections, and opportunities to progress to TB. In addition, the local differences in TB incidence likely overlaps with differences in the local distribution of social determinants of health (e.g., poverty, overcrowding, and housing conditions), which also influence TB epidemiology (*12*).

Our finding that localized transmission was associated with young age might be reflective of differences in the frequency and intensity of social activities across the course of life (*25*). Younger patients might have had more social connections and relationships with nonfamily members (*25*).

In addition, older patients might have progressed to having TB with non-genotype clustered strains from infections in the distant past (*26*). The large number of patients living in the same neighborhoods of another patient, and high proportion matching another patient by TB genotype, suggests targeted screening and treatment in high-burden neighborhoods might be cost-effective. Overall, a substantial proportion of patients (22%) resided at the same address as another patient; among these patients, 29% were matched by TB genotype. However, major differences occurred by geography. Among patients residing at the same address in Ghanzi, 50% were matched by genotyping, suggesting that household contact investigations in this district would be particularly effective at reducing transmission.

Social venue data suggested that community-based interventions might be effective for interrupting transmission. A substantial number of patients named the same places of worship (32%) or alcohol-related venues (30%) as another patient. Among persons naming the same alcohol-rated venue in Ghanzi, 57% were matched by TB genotype. These findings might help prioritize resources and guide effective strategies to interrupt *M. tuberculosis* transmission, such as intensified TB case finding in higher-burden geographic areas and targeted screening of frequently named social gathering venues.

Our results also highlight the need for using local data for local solutions. Comparative differences in spatial and genotypic clustering within and between communities reinforce the relative role of local factors that drive TB transmission and incidence. The proportion of patients attributed to localized transmission was higher in Ghanzi than in Gaborone. This finding suggests that, although TB transmission is a serious issue in both communities, a relatively higher proportion of TB cases might be caused by recent exposure to an infectious TB case-patient in Ghanzi than in Gaborone. Differences in population density and behavioral factors, such as smoking, drinking, and social mixing, highlight the potential impact of targeting interventions for these vulnerable populations. However, in settings that have prevalent endemic strains, genotype clustering might not be caused by recent transmission; higher resolution molecular characterization, such as whole-genome sequencing, might help further distinguish recent transmission from reactivation of highly prevalent, closely related strains.

The trend toward an inverse population- and individual-level association between HIV and localized transmission is consistent with findings from previous TB molecular epidemiologic studies in Africa and highlights the complex time-dependent interactions between the TB and HIV epidemics (*27*–*32*). At the population-level, Gaborone neighborhoods, which had the highest proportion of HIV–co-infected TB patients also demonstrated the lowest TB incidence, and HIV infection was negatively associated with localized transmission. HIV–co-infected TB patients progress more rapidly to active disease after *M. tuberculosis* infection and are generally less infectious and have higher mortality rates (*33*). As HIV care improves and antiretroviral therapy becomes more widely available, TB incidence among the HIV-infected persons decreases, leading to decreasing the rates of progression to active disease (*34*). Furthermore, being infected with HIV often means more visits to healthcare facilities in which TB screening is part of routine visits.

Our population-based design and multidisciplinary approach enables a high degree of confidence in our results and conclusions. However, major limitations need to be considered. First, although our study was multiyear and covered a broad geographic area, it is possible that some members of the transmission networks were missed (e.g., given a diagnosis before the study period or resided in areas not covered by the study, or refused enrollment) leading to genotype clustering misclassification. Also, not all enrolled TB patients produced sputum samples, and not all samples led to *M. tuberculosis* isolation or valid genotype results. This limitation might result in missed transmission links. Second, our molecular techniques characterized only part of the *M. tuberculosis* genome (*17*). It possible that genetic heterogeneity in loci not covered by this method might have been missed, resulting in misclassification (*17*). Moreover, the use of 1 isolate/patient, exclusion of mixed infections, missing data, and recall bias for naming potential transmission venues should also be acknowledged as potential limitations.

The Kopanyo Study adds to understanding of TB transmission dynamics in settings hyperendemic for TB and HIV by providing empirical data demonstrating the role of localized TB transmission during district-level epidemics. Thus, interrupting TB transmission in Botswana might warrant local solutions tailored for community differences and based on local epidemiology.

Appendix Additional information on population-based geospatial and molecular epidemiologic study of tuberculosis transmission dynamics, Botswana, 2012–2016.
