# Supplementary material for: Population-Based Geospatial and Molecular Epidemiologic Study of Tuberculosis Transmission Dynamics, Botswana, 2012–2016
Source: Emerg Infect Dis. 2021 Mar;27(3):835–44. doi: 10.3201/eid2703.203840 (PMC7920683; doi:10.3201/eid2703.203840)
Supplement: Appendix — Additional information on population-based geospatial and molecular epidemiologic study of tuberculosis transmission dynamics, Botswana, 2012–2016. [file 20-3840-Techapp-s1.pdf]

Article DOI: https://doi.org/10.3201/eid2703.203840

Population-Based Geospatial and Molecular Epidemiologic Study of Tuberculosis Transmission Dynamics, Botswana, 2012–2016

Appendix

Appendix Table. Bivariate analyses of demographic, socioeconomic, behavioral, and clinical characteristics associated with localized tuberculosis transmission, Botswana, 2012–2016*

Characteristic	Member of localized tuberculosis transmission, no. (%), n = 780	Not a member of localized tuberculosis transmission, no. (%), n = 1,016	χ^2	p-value
Sex				
M	436 (55.9)	555 (54.6)	0.29	0.591
F	344 (44.1)	461 (45.4)		
Age, y				
<u><</u>15	26 (3.3)	18 (1.8)	9.76	0.044
16–24	157 (20.1)	170 (16.7)		
25–40	418 (53.6)	558 (54.9)		
41–64	158 (20.3)	241 (23.7)		
<u>></u>65	21 (2.7)	29 (2.9)		
Resided in high-burden geographic area†				
Yes	291 (37.1)	181(17.8)	86.52	<0.001
No	489 (62.9)	835 (82.2)		
Excessive alcohol use‡				
Yes	160 (20.6)	205 (20.2)	0.03	0.861
No	620 (79.4)	811 (79.8)		
Current smoker				
Yes	250 (32.1)	220 (21.7)	24.72	<0.001
No	530 (67.9)	796 (78.3)		
Unemployed				
Yes	314 (41.6)	352(35.3)	5.94	0.015
No	466 (58.4)	664 (64.7)		
History of incarceration				
Yes	49 (6.3)	56 (5.5)	0.48	0.491
No	731 (93.7)	960 (94.5)		
HIV status				
Positive	366 (48.5)	570 (57.2)	14.91	0.001
Negative	414 (51.5)	446(42.8)		
Previous tuberculosis episode				
Yes	156 (20.0)	178(17.5)	1.79	0.181
No	624 (80.0)	838(82.5)		

*Localized transmission was defined by SaTScan (https://www.satscan.org)–identified geographic areas with a larger-than-expected rate of unique genotype clustering compared with all other culture-positive tuberculosis patients as the background rate; excludes 128 patients with valid genotype results and no residential address.
†Residing in a geographic area that had an estimated annualized tuberculosis incidence >305 patients/100,000 persons.
‡Five or more drinks per session in the previous 30 d or drinking on <u>></u>5 days in the previous 30 d.
